# Vigabatrin-Induced Encephalopathy in a 5.5-Month-Old Girl with Infantile Spasms due to Tuberous Sclerosis

**DOI:** 10.1155/2019/7249237

**Published:** 2019-08-25

**Authors:** Eleni Klinaki, Ioanna Argyri, Georgia Amountza, Gerina Ioannidou, Despoina Maritsi, Anastasia Garoufi, George Vartzelis

**Affiliations:** 2nd Pediatric Department of National and Kapodistrian University of Athens, Children's Hospital “P. & A. Kyriakou”, Athens, Greece

## Abstract

A 5.5-month-old female infant with tuberous sclerosis complex presented with infantile spasms and was treated with vigabatrin. As her condition did not improve, she was given adrenocorticotropic hormone (ACTH) intramuscularly which stopped the spasms and improved the electroencephalogram (EEG) abnormalities. However, she developed encephalopathy with apathy, drowsiness, and generalized slowing in the EEG. Discontinuation of vigabatrin quickly improved her symptoms and reversed the EEG slowing. A high index of suspicion is required in order to diagnose vigabatrin-induced encephalopathy, especially as the underlying disorders of these patients can be erroneously considered the cause of the observed encephalopathy.

## 1. Introduction

Infantile spasms (IS) is a serious epileptic disorder that often leads to developmental regression and life-long disabilities [1]. The single most common cause of IS is Tuberous Sclerosis Complex (TSC), a genetic neurocutaneous disorder that affects cellular differentiation and proliferation [2]. Vigabatrin is considered the first-line treatment for TSC-related IS with good overall rates of seizure control and cognitive outcome [3]. Herein, we present the case of a 5.5-month-old infant with TSC and IS who developed reversible encephalopathy following treatment with vigabatrin.

## 2. Case Report

A well-developed 5.5 month-old female infant was admitted in our department with semiology consistent with IS and confirmed on EEG testing which revealed hypsarrythmia. As she bore numerous hypomelanotic macules, brain imaging through Magnetic Resonance (MRI) was carried out revealing several brain hamartomas. She was diagnosed with TSC and was started on vigabatrin. Before initiation of vigabatrin, she was having more than five clusters of spasms per day. Despite her condition, she had reached the anticipated for her age, developmental milestones. Vigabatrin was administered with a starting dose of 50 mg/kg/d, and in two-week period, it was gradually increased to 120 mg/kg/d. The response to treatment was deemed unsatisfactory; therefore, synthetic adrenocorticotropic hormone (ACTH) was added in the treatment regimen and was administered intramuscularly. She remained hospitalized under close medical supervision and regular EEG follow-up. On the new treatment, her spasms gradually improved and the daily seizure count was greatly diminished. However, and despite the improvement in seizure control, she exhibited psychomotor regression along with signs and symptoms of encephalopathy. She was lethargic, unresponsive to acoustic and tactile stimuli, she was not smiling or rolling, and she exhibited head lag as well as head and upper limbs tremor. At that point, a repeat EEG revealed resolution of the hypsarrythmia, remained however significantly abnormal, and was dominated by diffuse slow delta rhythms of medium amplitude. A basic metabolic workup including ammonia, urine organic acids, and serum lactate and pyruvate was conducted with no abnormal findings. The administration of vigabatrin was stopped immediately, as it was considered a possible incriminating factor for the deterioration of the child's condition. Shortly after the discontinuation of vigabatrin, a substantial clinical improvement was observed and the infant returned to her previous normal developmental status, thus suggesting the encephalopathy was vigabatrin-induced. A repeat EEG showed overall improvement with normal background rhythms and occasional epileptic spikes.

## 3. Discussion

TSC is an autosomal dominant neurocutaneous genetic disorder of cellular differentiation and proliferation which variably affects the brain, skin, kidneys, heart, and other organs through the formation of hamartomas [4]. TSC is estimated to affect 25,000–40,000 individuals in the United States and 1-2 million individuals worldwide, with a prevalence of 1 in 6,000 live births [5]. Over 85% of patients with TSC are found to manifest mutations in TSC1 or TSC2, either through autosomal dominant inheritance, de novo mutations, or gonadal mosaicism [6].

The central nervous system is one of the most commonly affected systems in TSC. Epilepsy, neurocognitive dysfunction, and pervasive developmental disorders, such as autism, are perhaps the most devastating and therapeutically challenging manifestations of this condition. Seizures are the most common neurological disorder affecting up to 90% of individuals. They usually manifest during the first year of life in TSC patients and are associated with brain structural abnormalities. A variety of seizure types have been documented, including IS, simple partial, complex partial, and generalized tonic-clonic seizures, with IS being the most common initial seizure subtype [7]. Prompt treatment of IS is essential for an optimal cognitive outcome. Autism and intellectual disability later in life have been found to be associated with delays in treatment onset [8]. Over the past few decades, emerging clinical evidence has supported the use of vigabatrin as a first-line agent for TSC-related IS [3]. Vigabatrin is an antiepileptic drug that inhibits the catabolism of gamma-aminobutyric acid (GABA) by irreversibly inhibiting GABA transaminase [9]. It is generally well-tolerated with the most common adverse events being sedation and myoclonias. Rare serious side effects are peripheral visual field defects and vigabatrin-induced encephalopathy. The latter is a fairly uncommon complication of unknown aetiology with only small numbers of cases been reported in the literature [10–12]. On occasions, it has been associated with MRI signal changes although these usually occur without any clinical symptoms and resolve even if treatment is continued [13, 14]. From five patients reported to date in the literature with IS and vigabatrin-related encephalopathy, two were suffering underlying white matter disorders, one with Alexander disease, and one with myelination delay [12–15]. The other three patients developed the encephalopathy in the absence of a preexisting white matter disorder and had however received synchronous treatment with ACTH which could be relevant to the pathogenesis of the condition [16, 17]. In our case, the close temporal association between the clinical and electrophysiological improvement and the withdrawal of vigabatrin, together with the fact that no other parameter regarding the treatment regimen was altered, strongly suggests that vigabatrin was most likely the incriminating agent. Our literature search did not reveal any TSC patients on vigabatrin therapy known to have developed this rare complication. The fact that vigabatrin is considered the first-line agent for the treatment of TSC-related IS, combined with the knowledge that TSC epilepsy can result to encephalopathy and developmental regression, make it even more challenging for clinicians to attribute the encephalopathic symptoms to the therapy alone. It is however crucial to recognize the culprit, as misdiagnosing the condition can lead to significant iatrogenic morbidity.

In conclusion, vigabatrin-induced encephalopathy is a rare complication that can affect infants with IS. It can even occur when the spasms are secondary to TSC where vigabatrin is considered the first-line therapy; therefore, a high index of suspicion for this condition has to be maintained.

## References

[1] Pellock J. M., Hrachovy R., Shinnar S. (2010). Infantile spasms: a U.S. consensus report. *Epilepsia*.

[2] Watanabe K. (1998). West syndrome: etiological and prognostic aspects. *Brain and Development*.

[3] Overwater I. E., Bindels-de Heus K., Rietman A. B. (2015). Epilepsy in children with tuberous sclerosis complex: chance of remission and response to antiepileptic drugs. *Epilepsia*.

[4] Wang S., Fallah A. (2014). Optimal management of seizures associated with tuberous sclerosis complex: current and emerging options. *Neuropsychiatric Disease and Treatment*.

[5] Roach E. S. (2016). Applying the lessons of tuberous sclerosis: the 2015 hower award lecture. *Pediatric Neurology*.

[6] Crino P. B., Nathanson K. L., Henske E. P. (2006). The tuberous sclerosis complex. *New England Journal of Medicine*.

[7] Orlova K. A., Crino P. B. (2010). The tuberous sclerosis complex. *Annals of the New York Academy of Sciences*.

[8] Bombardieri R., Pinci M., Moavero R., Cerminara C., Curatolo P. (2010). Early control of seizures improves long-term outcome in children with tuberous sclerosis complex. *European Journal of Paediatric Neurology*.

[9] Aneja S., Sharma S. (2013). Newer anti-epileptic drugs. *Indian Pediatrics*.

[10] Vega Y. H., Kaliakatsos M., U-King-Im J.-M., Lascelles K., Lim M. (2014). Reversible vigabatrin-induced life-threatening encephalopathy. *JAMA Neurology*.

[11] Tekgul H., Serdarolu G., Karapinar B. (2006). Vigabatrin caused rapidly progressive deterioration in two cases with early myoclonic encephalopathy associated with nonketotic hyperglycinemia. *Journal of Child Neurology*.

[12] Haas-Lude K., Wolff M., Riethmuller J., Niemann G., Krageloh-Mann I. (2000). Acute encephalopathy associated with vigabatrin in a six-month-old girl. *Epilepsia*.

[13] Dracopoulos A., Widjaja E., Raybaud C., Westall C. A., Snead O. C. (2010). Vigabatrin-associated reversible MRI signal changes in patients with infantile spasms. *Epilepsia*.

[14] Thelle T., Gammelgaard L., Hansen J. K., Østergaard J. R. (2011). Reversible magnetic resonance imaging and spectroscopy abnormalities in the course of vigabatrin treatment for West syndrome. *European Journal of Paediatric Neurology*.

[15] Horton M., Rafay M., Del Bigio M. R. (2009). Pathological evidence of vacuolar myelinopathy in a child following vigabatrin administration. *Journal of Child Neurology*.

[16] Pearl P. L., Vezina L. G., Saneto R. P. (2009). Cerebral MRI abnormalities associated with vigabatrin therapy. *Epilepsia*.

[17] Dill P., Datta A. N., Weber P., Schneider J. (2013). Are vigabatrin induced T_2_ hyperintensities in cranial MRI associated with acute encephalopathy and extrapyramidal symptoms?. *European Journal of Paediatric Neurology*.

